# Decoding Dental Stem Cell Aging: Mechanisms, Therapeutic Strategies, and Beyond

**DOI:** 10.1002/advs.202504969

**Published:** 2025-10-17

**Authors:** Xinyuan Zhao, Yunfan Lin, Pei Lin, Ye Lu, Jiarong Zheng, Xu Chen, Zihao Zhou, Li Cui

**Affiliations:** ^1^ Stomatological Hospital School of Stomatology Southern Medical University Guangzhou Guangdong 510280 China; ^2^ Department of Dentistry The First Affiliated Hospital Sun Yat‐sen University Guangzhou 510080 China; ^3^ School of Dentistry University of California, Los Angeles Los Angeles CA 90095 USA

**Keywords:** aging, dental stem cells, regenerative medicine

## Abstract

Dental stem cells (DSCs) hold immense potential in regenerative medicine due to their unique properties, including superior proliferative and differentiation capacities, robust immunomodulatory functions, and resilience to aging. However, the aging process profoundly impairs their functionality, diminishing their regenerative potential and limiting their clinical utility. This review provides a systematic examination of the mechanisms underlying DSC aging, focusing on disrupted signaling pathways, metabolic dysregulation, and epigenetic modifications, as well as the regulatory roles of non‐coding RNAs and critical proteins. It further investigates the key intrinsic and extrinsic factors driving this process, offering a comprehensive perspective on the interplay between cellular and systemic influences. Building on this foundation, the review explores innovative strategies to mitigate age‐related decline in DSCs, emphasizing approaches that target the extracellular matrix, mitochondrial dysfunction, and key molecular pathways. Finally, it addresses the challenges in translating these findings into clinical applications, such as inter‐individual variability and systemic influences, and advocates for multidisciplinary approaches to enhance therapeutic outcomes. Collectively, this review provides a critical framework for advancing the clinical translation of DSC‐based therapies, with broader implications for regenerative medicine in aging contexts.

## Graphical abstract

Dental stem cell (DSC) aging involves genomic instability, mitochondrial dysfunction, telomere attrition, and epigenetic alterations, leading to impaired proliferation, reduced differentiation potential, and pro‐inflammatory secretory activity. These processes drive cellular senescence and compromise regenerative and immunomodulatory functions, thereby limiting therapeutic efficacy. Defining the molecular basis of DSC aging not only clarifies the mechanisms underlying their decline but also informs strategies for rejuvenation, quality control, and patient stratification, which are essential for advancing their clinical translation in tissue repair and disease treatment.

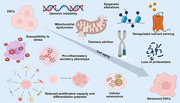

## 1 Introduction

Dental stem cells (DSCs) represent a unique subset of mesenchymal stem cells (MSCs) with distinctive biological and clinical attributes.^[^ ^1^ ^]^ Compared with non‐dental MSCs such as bone marrow‐ or adipose‐derived MSCs, DSCs demonstrate superior proliferative capacity and clonogenicity, enhanced multipotent differentiation potential toward odontogenic, neurogenic, and angiogenic lineages, as well as stronger immunomodulatory effects, including the regulation of T‐cell responses and macrophage polarization.^[^ ^2^, ^3^, ^4^ ^]^ They also display delayed senescence and heightened resistance to oxidative stress, which improve their long‐term stability in culture. These intrinsic advantages have been substantiated in translational studies, where DSCs have achieved promising outcomes not only in dental tissue regeneration, including pulp‐dentin complex and periodontal repair, but also in broader clinical contexts such as craniofacial bone reconstruction, corneal surface restoration, neural injury repair, and autoimmune disease modulation.^[^ ^1^, ^5^, ^6^, ^7^ ^]^ Such evidence underscores that DSCs not only surpass conventional MSCs at the mechanistic level but also hold distinct superiority in clinical applications, making them an attractive cell source for regenerative medicine.

“Aging” refers to the progressive decline in physiological integrity and function at the organismal and tissue levels, driven by cumulative genetic, metabolic, and environmental influences. In contrast, “senescence” denotes a state of irreversible cell cycle arrest at the cellular level, typically accompanied by characteristic features such as telomere shortening, DNA damage, and the senescence‐associated secretory phenotype (SASP). Cellular senescence is a complex biological process characterized by the progressive decline in cellular function, ultimately leading to reduced tissue regeneration and organismal homeostasis. Key hallmarks of aging include genomic instability, telomere attrition, mitochondrial dysfunction, loss of proteostasis, epigenetic alterations, and deregulated nutrient sensing. These changes result in diminished cellular proliferation, increased susceptibility to stress, and the onset of senescence, a state of irreversible cell cycle arrest accompanied by a pro‐inflammatory secretory phenotype. senescence impacts not only individual cells but also the surrounding microenvironment, contributing to systemic dysfunction and the decline in tissue regeneration^[^ ^8^, ^9^, ^10^ ^]^

**Figure 1:**
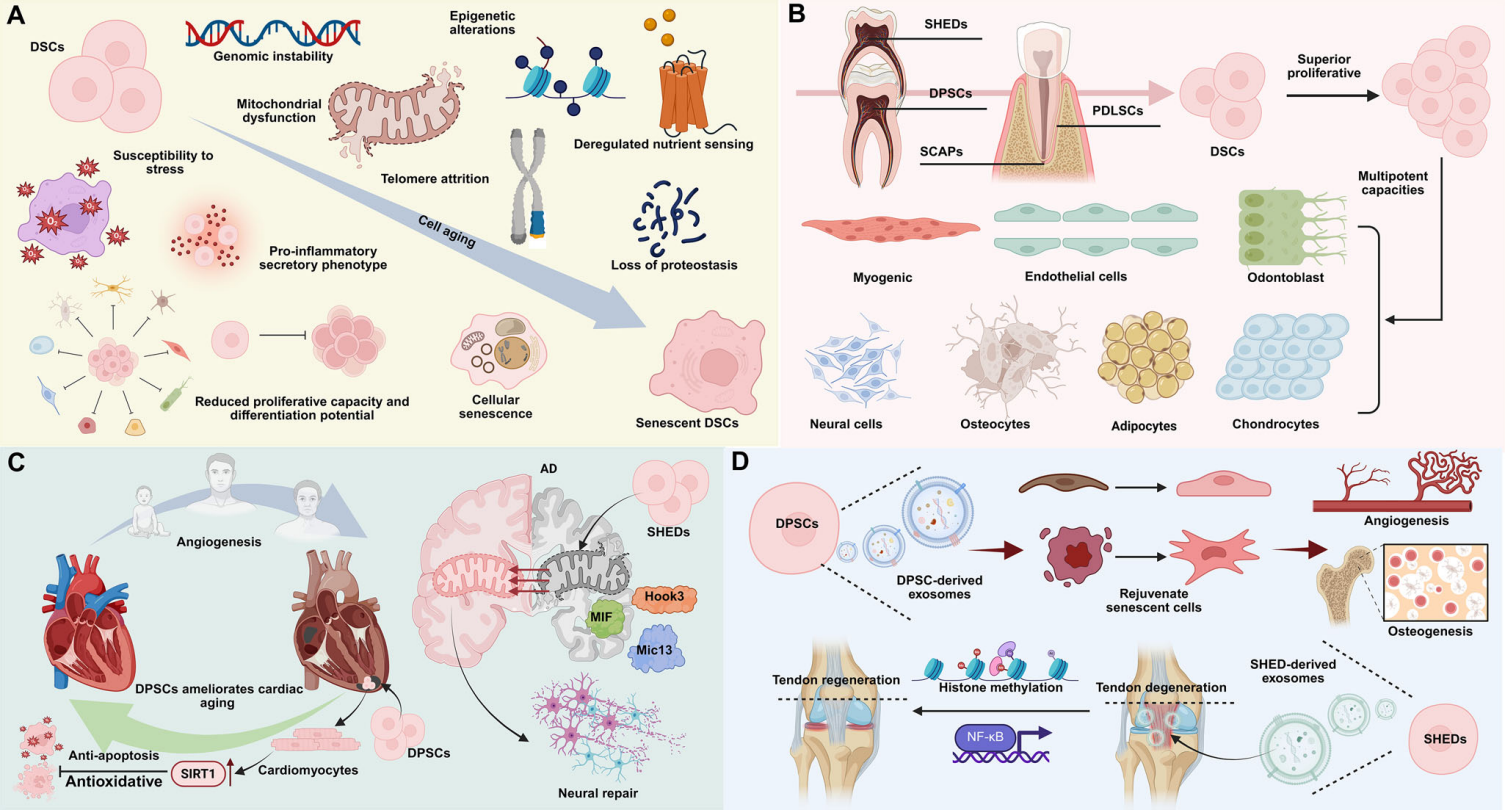
Overview of DSCs and their extracellular vesicles. A) Aging‐related alterations in DSCs, including genomic instability, mitochondrial dysfunction, telomere attrition, and loss of proteostasis, lead to reduced proliferative capacity and differentiation potential. These changes, along with a pro‐inflammatory secretory phenotype, ultimately drive cellular senescence in DSCs. B) DSC are a unique subset of MSCs with significant potential in regenerative medicine, exhibiting superior proliferative capacity and multipotent differentiation abilities. C) Transplanted DPSCs migrate to the heart, differentiate into cardiomyocytes, and enhance SIRT1 expression, exerting antioxidative, anti‐senescent, and anti‐apoptotic effects. Additionally, SHED treatment alleviates AD pathology, targeting mitochondrial dysfunction via key mitochondrial proteins such as Hook3, Mic13, and MIF. D) Extracellular vesicles derived from DSCs promote tendon regeneration, rejuvenate senescent cells, and stimulate osteogenesis and angiogenesis primarily through the modulation of NF‐kB signaling and histone methylation.

DSCs, despite their robust regenerative potential, are not exempt from these senescence processes. As they age, their functionality is significantly impaired, with reduced proliferative capacity, differentiation potential, and immunomodulatory efficiency. Mechanistically, this decline is linked to disrupted signaling pathways, metabolic imbalances, and epigenetic dysregulation. Furthermore, external factors such as chronic inflammation and metabolic disorders exacerbate these intrinsic changes, amplifying the effects of aging^[^ ^11^, ^12^ ^]^ (**Figure** **1**A). These combined challenges highlight the critical need for innovative approaches to preserve the regenerative capacity of DSCs and ensure their effective clinical application.

In this review, we present the first comprehensive and systematic analysis of the relationship between DSCs and aging, highlighting key advances and challenges in this emerging field. First, we provide a detailed synthesis of the molecular mechanisms regulating DSC aging, including disrupted signaling pathways, metabolic dysregulation, and epigenetic modifications. Second, we explore the diverse factors influencing DSC aging. Third, we evaluate effective strategies for mitigating the impact of aging on DSCs. Finally, we discuss the challenges and future perspectives in the field, emphasizing the need for interdisciplinary approaches and translational research to address age‐related limitations and unlock the full potential of DSCs in regenerative medicine. This review integrates current knowledge while offering new insights and directions, serving as a critical foundation for advancing research in DSC senescence.

## 2 Therapeutic Potential of DSCs and Their Extracellular Vesicles in Regenerative Medicine and Aging‐Related Disorders

DSCs are uniquely advantageous in regenerative medicine due to their superior proliferative, multipotent, immunomodulatory properties, and enhanced resistance to senescence, surpassing those of non‐dental MSCs in various aspects. Dental pulp stem cells (DPSCs) exhibit superior resistance to senescence and apoptosis compared to non‐dental MSCs, maintaining higher proliferation, enhanced osteogenesis, and lower inflammatory sensitivity. DPSCs outperform bone marrow MSCs in lipopolysaccharide‐induced senescence and promote periodontal regeneration in vivo.^[^ ^11^, ^13^ ^]^ Likewise, DPSCs exhibit lower intracellular ROS levels and reduced β‐gal expression compared to umbilical cord‐derived stem cells at higher passages, indicating superior resistance to replicative senescence^[^ ^14^ ^]^ (Figure 1B).

DSCs encompass several distinct populations isolated from different dental tissues, each possessing unique developmental origins and biological characteristics. DPSCs, first identified within the dental pulp of permanent teeth, exhibit high proliferative capacity and multilineage differentiation potential, particularly toward odontogenic, osteogenic, and neurogenic lineages.^[^ ^4^ ^]^ Periodontal ligament stem cells (PDLSCs) are derived from the periodontal ligament and are specialized in generating cementum/periodontal ligament‐like structures, making them highly valuable for periodontal tissue regeneration.^[^ ^15^ ^]^ Stem cells from human exfoliated deciduous teeth (SHED) originate from the pulp of naturally shed deciduous teeth and display superior proliferative and clonogenic potential, with remarkable neurogenic and angiogenic differentiation capacity, reflecting their immature developmental stage.^[^ ^16^ ^]^ Stem cells from the apical papilla (SCAP), isolated from the root apical papilla of developing permanent teeth, are characterized by robust self‐renewal and mineralization abilities, contributing to root dentin formation and root development.^[^ ^17^ ^]^ In addition to these well‐established populations, other DSC sources have been identified, such as dental follicle progenitor cells (DFPCs) from the tooth germ and gingiva‐derived mesenchymal stem cells (GMSCs) from gingival connective tissue, both of which further broaden the spectrum of DSCs available for regenerative applications.^[^ ^18^, ^19^ ^]^

Growing evidence indicates that DSCs and their derivatives exert therapeutic effects across multiple aging‐related conditions through common mechanisms such as antioxidation, anti‐senescence, and mitochondrial protection. For example, systemic transplantation of DPSCs alleviates cardiac aging in D‐galactose‐induced rats by differentiating into cardiomyocyte‐like cells, upregulating SIRT1, and improving connexin‐43 expression, ultimately restoring cardiac function ^[^ ^20^ ^]^ (Figure 1C). In the nervous system, DPSCs have been shown to differentiate into dopaminergic‐like cells, improving motor function and reducing oxidative stress in Parkinson's disease models, while SHED transplantation ameliorates Alzheimer's pathology in SAMP8 mice by rescuing mitochondrial dysfunction and modulating key mitochondrial proteins.^[^ ^21^ ^]^ In metabolic organs, SHED delivery reversed liver proteomic profiles from senescent to youthful states, restored hepatocyte function, and enhanced mitochondrial activity, underscoring their potential in systemic rejuvenation.^[^ ^22^ ^]^

Beyond whole‐cell transplantation, extracellular vehicles (EVs) and secretome derived from DSCs reproduce many of these anti‐aging effects. DPSC‐derived small EVs protect against irradiation‐induced salivary gland senescence by reducing inflammatory cytokine expression and restoring acinar cell function.^[^ ^23^ ^]^ In regenerative contexts, engineered DPSC‐derived exosomes loaded with cordycepin (CY@D‐exos) rejuvenate senescent bone marrow MSCs and endothelial cells, enhancing osteogenesis and angiogenesis via NRF2 activation.^[^ ^24^ ^]^ Moreover, Exosomes secreted by stem cells from SHED exhibit pronounced anti‐aging effects on aged tendon stem/progenitor cells (AT‐SCs). They preserve the tenogenic differentiation potential of AT‐SCs through epigenetic regulation, notably by modulating histone methylation patterns and suppressing NF‐κB signaling. This regulation is mediated by specific control of repressive histone marks such as H3K9me3 and H3K27me3. In experimental models of aged tendons, both systemic administration and local delivery of SHED‐derived exosomes attenuated tendon degeneration, reduced cellular senescence, and enhanced regenerative capacity ^[^ ^25^, ^26^ ^]^ (Figure 1D). Taken together, these studies highlight that DSCs and their secreted products not only target organ‐specific aging phenotypes but also share convergent anti‐aging mechanisms, offering broad therapeutic potential across cardiovascular, neurological, hepatic, musculoskeletal, and integumentary systems.

## 3 The Impact of Aging on the Functionality of DSCs

“Young” DSCs refer to cells isolated from donors in childhood or early adulthood, typically exhibiting long telomeres, high proliferative capacity, and low senescence‐associated β‐gal activity.^[^ ^27^, ^28^ ^]^ In contrast, “aged” DSCs denote cells derived from older donors, or cells that have undergone long‐term in vitro passaging, and are characterized by shortened telomeres, reduced proliferation, and increased senescence markers (e.g., p16, p21, γ‐H2AX).^[^ ^29^ ^]^ Aging exerts profound effects on DSCs, progressively impairing their proliferative, regenerative, and immunomodulatory capacities. These changes can be broadly categorized into phenotypic alterations, dysregulated signaling pathways, and inter‐individual heterogeneity. Human dental pulp cells undergoing replicative or stress‐induced premature senescence display enhanced β‐galactosidase activity, ROS accumulation, and altered expression of inflammatory mediators.^[^ ^30^ ^]^ Autophagic activity is also altered, with upregulation of LC3 and Beclin 1 and increased autophagic vacuoles in senescent DPSCs. Telomere shortening, a hallmark of cellular aging, drives genomic instability and replicative senescence, ultimately restricting functional longevity.^[^ ^31^ ^]^ Notably, using scanning ion conductance microscopy, extracellular topography and surface charge distribution of DPSCs were mapped, revealing a significant increase in negative surface charge during cellular senescence. This approach offers precise insights into surface charge alterations associated with aging.^[^ ^32^ ^]^ Similar phenotypic declines are observed in PDLSCs, which exhibit reduced proliferation, osteogenic/adipogenic potential, and immunosuppressive activity, alongside increased apoptosis and downregulation of Runx2, ALP, and COL1A1.^[^ ^33^ ^]^ Notably, aging impairs the adipogenic and osteogenic differentiation of gingival GMSCs but does not affect neurogenesis. Increased p53 and SIRT1 expression with age enhances tumor suppression via apoptosis and autophagy. Despite these changes, GMSCs largely preserve their immunoregulatory functions, demonstrated by their capacity to suppress T‐cell proliferation, limit neutrophil infiltration, and downregulate pro‐inflammatory cytokines (TNF‐α, TGF‐β, IFN‐γ) in a mouse model of acute lung injury. Alongside their regenerative properties, these features highlight the therapeutic promise of GMSCs for inflammatory disorders such as ARDS and COVID‐19.^[^ ^34^ ^]^

Several signaling pathways mediate the functional decline of DSCs with age. In DPSCs, reduced osteogenic potential is linked to attenuated parathyroid hormone (PTH)–PTH1R signaling, whereas juvenile SHEDs display higher PTH secretion, increased mineralization, and elevated osteogenic marker expression compared to older DPSCs.^[^ ^35^ ^]^ Neurogenic differentiation also declines with age, yet can be partially restored through activation of Wnt/β‐catenin signaling, underscoring its role in age‐dependent regenerative potential.^[^ ^36^ ^]^ In PDLSCs, diminished responsiveness to PTH and osteoblast‐specific markers reflects compromised hormonal sensitivity.^[^ ^37^ ^]^ In GMSCs, age‐associated upregulation of p53 and SIRT1 enhances apoptosis and autophagy, representing a tumor‐suppressive adaptation at the cost of regenerative potential.^[^ ^34^ ^]^ Additionally, aging alters the immunomodulatory properties of DPSCs and DP‐MSCs: younger cells enhance Th17 activity through IL‐6, IL‐17a, and HGF expression, whereas older cells promote Treg expansion and TGF‐β expression, revealing age‐dependent shifts in immune regulation.^[^ ^38^ ^]^ Consistently, aging also affects PDLSC‐mediated immunosuppression of peripheral blood mononuclear cells (PBMCs), reducing T lymphocyte proliferation, CD28 expression, and cytokine secretion (IL‐2, IFN‐γ) while increasing CD95 and TNF‐β. PDLSCs suppress T cell proliferation more strongly in older PBMCs, reflecting age‐dependent immunosuppressive dynamics.^[^ ^39^ ^]^

Aging effects on DSCs are not uniform but vary across donors, tissues, and microenvironments. Proliferative and regenerative heterogeneity in DPSCs correlates with telomere length and CD271 expression: high‐proliferative subsets retain stemness and multipotency, whereas low‐proliferative, CD271⁺ subsets exhibit adipogenic bias and impaired differentiation. Similarly to DPSCs, aging reduces the proliferation, differentiation potential, and immunosuppressive capacity of PDLSCs while increasing apoptosis. These changes are associated with altered expression of key genes and proteins, including decreased Runx2, ALP, and COL1A1, and increased CCND3. Importantly, their immunoregulatory capacity is compromised, as reflected by a weakened ability to suppress T‐cell proliferation, inhibit dendritic cell maturation, promote macrophage M2 polarization, and secrete anti‐inflammatory mediators such as IL‐10, TGF‐β, IDO, and PGE2.^[^ ^33^ ^]^ Microenvironmental cues also shape age‐related changes. Conditioned medium from juvenile DPSCs enhances adult DPSC proliferation, while adult DPC‐CM augments osteogenic potential in juvenile cells.^[^ ^40^ ^]^ In PDLSCs, young cell–derived factors restore the regenerative capacity of aged cells, whereas exposure to aged conditioned medium impairs the differentiation of young cells.^[^ ^41^ ^]^ These findings highlight the reciprocal influence between intrinsic aging programs and extrinsic niche signals in determining DSC functionality. Collectively, aging drives phenotypic senescence, disrupts key signaling pathways, and reveals heterogeneity shaped by intrinsic and extrinsic factors. Understanding these multilayered changes provides a foundation for developing targeted strategies to preserve or restore the regenerative capacity of DSCs in aging populations.

## 4 Key Factors Affecting the Senescence of DSCs

**Figure 2:**
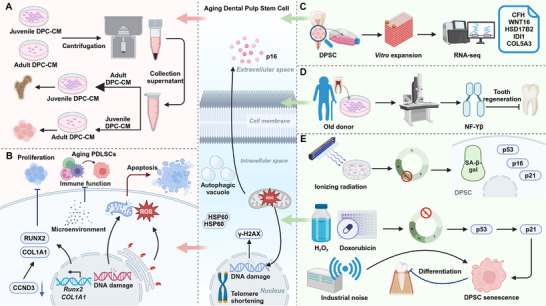
Impact of aging on DPSC function and key regulatory factors. A) CM from juvenile and adult DPCs influences DPSC functionality. Juvenile DPC‐CM enhances proliferation and osteogenesis, whereas adult DPC‐CM alters the microenvironment, impairing regenerative potential. B) Aging PDLSCs undergo microenvironmental shifts, leading to immune suppression, apoptosis, and increased ROS levels, which contribute to DNA damage, reduced RUNX2 and COL1A1 expression, and elevated CCND3, collectively impairing function. C) In vitro expansion of DPSCs followed by RNA‐seq reveals age‐related transcriptional changes, including dysregulation of Wnt/β‐catenin signaling, which reduces regenerative capacity. D) DPSCs from aged donors exhibit increased senescence markers and reduced NF‐κB activity, leading to diminished tooth regeneration potential. E) External stressors such as ionizing radiation, H_2_O_2_, doxorubicin, and industrial noise accelerate DPSC senescence by inducing p53/p21 signaling, thereby impairing differentiation.

The senescence of DSCs can be triggered by factors such as prolonged culture, high passage numbers, donor aging, and exposure to ionizing radiation. These factors contribute to replicative senescence, oxidative stress, DNA damage, and diminished regenerative capacity, ultimately compromising their therapeutic potential (**Figure** **2**). For instance, extended in vitro expansion induces senescence in DPSCs, reducing proliferative capacity and osteogenic‐lipogenic differentiation potential. Senescence correlates with decreased expression of CD73, CD90, and CD105, while RNA sequencing identifies CFH, WNT16, HSD17B2, IDI1, and COL5A3 as potential regulators of this process.^[^ ^42^ ^]^ Similarly, prolonged passaging of DPSCs induces morphological abnormalities, including larger cell and nuclear sizes, reduced proliferative capacity, and slower growth in late passages, which are also characterized by higher collagen expression and lower osteocalcin levels.^[^ ^43^ ^]^Additionally, prolonged cultivation of SHEDs reveals aging‐associated declines in proliferation, chondrogenic, and osteogenic potential, alongside increased adipogenic differentiation and reduced autophagic capacity. Metabolic shifts, including altered fatty acid composition, transition SHEDs from anti‐inflammatory to proinflammatory pathways.^[^ ^44^ ^]^ Moreover, prolonged culture of DFCs induces cellular senescence, marked by reduced proliferation, increased cell size, and senescence‐associated β‐galactosidase activity. Senescence impairs osteogenic differentiation, suppressing RUNX2 and OPN expression and biomineralization.^[^ ^45^ ^]^

Donor age is a factor influencing the functionality and regenerative potential of DSCs. DPSCs from young donors exhibit superior proliferation and differentiation potential compared to those from old donors, with significant differences in lncRNA and mRNA expression profiles. Pathways related to the cell cycle and RNA transport, along with core regulators like nuclear transcription factor Y subunit β, were implicated in DPSC senescence, highlighting donor age as a critical factor in tooth regeneration.^[^ ^46^ ^]^ A hydroxyurea‐induced senescence model in DPSCs revealed age‐associated changes, including increased senescence markers, reduced proliferation and differentiation, G0/G1 arrest, elevated apoptosis, and higher ROS levels. Young donor DPSCs showed greater resistance to apoptosis and enhanced DNA repair via non‐homologous end‐joining.^[^ ^47^ ^]^ Similarly, PDLSCs from older donors exhibit reduced colony‐forming efficiency, proliferation, and osteogenic differentiation, alongside increased senescence and apoptosis. Markers of osteogenic potential, including Runx‐2, COL‐1, and ALP, decline with age, highlighting the diminished regenerative capacity of PDLSCs during aging.^[^ ^48^ ^]^ Additionally, PDLSCs from older donors exhibit reduced proliferation, osteogenic differentiation, and pluripotency‐associated transcription factor expression, alongside increased senescence. Aged PDLSCs form cell sheets with diminished extracellular matrix (ECM) protein production and lower osteogenic gene expression, impairing regenerative potential. These age‐related changes highlight the importance of considering donor age in cell sheet engineering and periodontal regenerative therapies.^[^ ^49^ ^]^ Interestingly, the secretomes of SHEDs and young DPSCs contain higher growth factor levels and fewer pro‐inflammatory cytokines compared to those from old permanent teeth, correlating with superior osteogenic and chondrogenic differentiation.^[^ ^50^ ^]^

In addition to prolonged culture and donor age, a number of exogenous stimuli can promote DSC aging. DPSCs exposed to ionizing radiation undergo stress‐induced premature senescence without apoptosis, marked by G2 phase arrest, SA‐β‐gal positivity, and upregulation of p53, p21, and p16.^[^ ^51^ ^]^ Similarly, ionizing radiation induces stress‐induced premature senescence in MSCs from bone marrow and periodontal ligament, characterized by p53 activation, elevated p21 expression, and G2 phase arrest.^[^ ^52^ ^]^ In addition, ionizing radiation induces premature differentiation of DPSCs into odonto‐/osteoblast lineages, compromising their immature state critical for tissue repair. Matrix calcification was observed at early stages, depending on CD146 expression levels, highlighting radiation's impact on stem cell plasticity and its potential implications for tissue regeneration in cancer therapies.^[^ ^53^ ^]^ Moreover, p‐Cresol induces cellular senescence in DPSCs by reducing proliferation, increasing senescence markers, and promoting inflammation. It disrupts the cell cycle, elevating Bax and reducing Bcl‐2, while impairing odontoblast differentiation, as shown by decreased DSPP, DMP1, and osterix expression.^[^ ^39^ ^]^ Notably, H_2_O_2_, doxorubicin, and UV irradiation effectively induce senescence in DPSCs, characterized by G1 phase arrest, increased p21 and BTG1 expression, and decreased CCND1 expression via the p53‐p21 pathway. UV exposure induces senescence rapidly, underscoring the need for minimized fluorescent and UV light exposure and low‐oxygen culture conditions to prevent premature DPSC senescence.^[^ ^54^ ^]^ Exposure to industrial noise induces morphological changes in the dental pulp, significantly reducing pulp cell density after 3 months in rats, resembling age‐related alterations in the pulp‐dentin complex. While prolonged exposure (7 months) showed no further differences, these findings suggest industrial noise as a potential accelerator of premature dental aging. In this study, the noise stimulus was objectively characterized by analyzing its frequency spectrum and amplitude distribution, ensuring reproducible exposure parameters for the experimental model.^[^ ^55^ ^]^

## 5 Mechanisms Driving Dental DSC Aging

Understanding the mechanisms underlying DSC aging is critical for advancing regenerative medicine and developing effective interventions to mitigate age‐related functional decline. These mechanisms not only govern the biological processes driving cellular senescence but also provide insights into potential therapeutic targets to restore stem cell function. In this section, we systematically explore the pathways and regulatory networks influencing DSC aging, highlighting their implications for both fundamental biology and clinical applications (**Table** **1**).

**Table 1:** Mechanisms of DSC aging.

Cell Type	Regulatory Factor	Key Target/Pathway	Mechanism	Impact on Senescence	Refs.
DFCs	AMPK	/	↓ Autophagy and glycolysis	Promoting senescence via metabolic shifts	[56]
PSCs	β‐catenin	Wnt/β‐catenin pathway	↑ β‐catenin and Wnt1	Promotion of cellular aging in hyperglycemic state	[57]
DFCs	Wnt5A	WNT pathway	/	Promoting a senescent‐like phenotype	[58]
hPDLSCs	YAP	Hippo‐YAP and ERK pathways	↑ P‐MEK, P‐ERK, P‐P90RSK ↓ Bcl‐2	Inhibition of DSC senescence, and apoptosis, whereas promoting proliferation	[59]
DFCs	RUNX2	ERK signaling pathway	↓ Senescence‐associated gene and protein	Promoting DSC senescence	[60]
hDPCs	DAPT	Notch signaling pathway	Influence metabolic enzyme levels and gene expression	Promoting cellular senescence	[61]
PDLSCs	TGF‐β1	TGF‐β signaling pathway	↑ β‐galactosidase, ROS and p16/ p21	Affecting the cellular aging process	[62]
DPSCs	ILK	mTOR signaling pathway	↑AKT/mTOR/STAT1 pathway ↓Senescence‐related genes	Decreasing SA‐β‐gal‐positive cells to alleviate aging	[63]
DPCs	miR‐433	GRB2 signaling pathway RAS‐MAPK signaling pathway	↓ Proliferation and mineralization ↑ Apoptosis	Enhancing DPC repair and regeneration in aging	[64]
DPSCs	Hsa‐miR‐6724‐5p	Dopaminergic synapse pathway	↑ mRNAs and lncRNAs	Promotion of senescence via modulation of dopaminergic synapse‐related genes	[65]
DPSCs	miR‐152	/	↓ SIRT7	Development of a senescent phenotype	[67]
DPSCs	miR‐152	CCNA2	↓ ROS accumulation, p53, p21, and p16	Resistance to aging	
SCAPs	miR‐141‐3p	YAP	/	Delaying senescence	[68]
DPSCs	Alu	Methylation	↑ Hypermethylated CpG dinucleotides	Regulating cellular senescence through pathway control of methylation levels	[69]
DPSCs	MSX2	ROR2/MSX2/NSUN2 axis	Modulating the phosphorylation and ubiquitination levels	Influencing cellular senescence	[70]
DPSCs	KDM3A KDM4C	H3K9 demethylation	↑ DNA damage response	Exacerbating cellular senescence	[71]
PDLSCs	AGEs	AGE/RAGE pathway	↓ KDM6B/Wnt feedback loop	Regulating cellular senescence	[72]
PDLSCs	HDAC6	p27Kip1 acetylation	/	Accelerating senescence and impairs stem cell activities	[73]
DPSCs	Barx1	TGF‐β pathway	Metabolic regulation	Cellular senescence inducing	[77]
PDLSCs	SIRT3	LRPPRC deacetylation	↑ Oxidative phosphorylation ↑ Oxidative stress	Activation of SIRT3 delays senescence	[79]
DPSCs	ROCK1	SIRT7/ROCK1 axis	↓ SIRT7 and TERT	Affecting cell senescence	[80]
DPSCs	ROR2	ROR2/STK4‐FOXO1/SMS1 axis	↑ OXO1 nuclear translocation ↑ SMS1 repression and SM production	Regulation of DPSC senescence	[81]
PDLSCs	PB2	NF‐κB signaling pathway	↓ Inflammation ↑ Antioxidant enzyme	Reducing senescence‐associated proteins and genes	[82]
PDLSCs	LRP5	mTOR signaling	↑ RUNX2 ↑ Collagen type I and β‐catenin	Restoring osteogenic capacity in aged PDLSCs	[83]

### 5.1 Disrupted Signaling Pathways

Cellular senescence in DSCs is a complex process influenced by disrupted signaling pathways that regulate proliferation, differentiation, and survival. For instance, cellular senescence in DFCs involves disrupted AMPK signaling, altered autophagy, and metabolic shifts. AMPK inhibition promotes senescence by impairing autophagy, while prolonged activation with metformin or AICAR paradoxically induces senescence via reduced autophagy and a glycolytic phenotype. These findings highlight a dual role of AMPK and mitochondrial dysfunction in driving senescence in DFCs.^[^ ^56^ ^]^ In addition, hyperglycemia accelerates dental pulp cell aging by reducing proliferation, increasing senescence markers, and upregulating Wnt/β‐catenin signaling. β‐Catenin activation exacerbates, while its inhibition mitigates, cellular aging in hyperglycemic conditions.^[^ ^57^ ^]^ Likewise, cellular senescence impairs osteogenic differentiation in DFCs, coinciding with reduced Wnt5A expression. Wnt5A inhibition by siRNA decreased cell proliferation and viability while increasing senescence and cell death, but had limited impact on osteogenic differentiation. These findings suggest Wnt5A plays a critical role in maintaining DFC viability during proliferation and differentiation, particularly under senescent conditions.^[^ ^58^ ^]^ Notably, activated YAP promotes proliferation and delays senescence in PDLSCs by increasing G2/M phase progression and reducing apoptosis through upregulation of P‐MEK, P‐ERK, and P‐P90RSK, while suppressing pro‐apoptotic Bcl‐2 family proteins. YAP translocation to the nucleus further enhances these effects, underscoring its role in maintaining PDLSC regenerative potential via hippo‐YAP and ERK signaling pathways.^[^ ^59^ ^]^ In contrast, RUNX2 mutations delay DFC senescence by suppressing ERK signaling, promoting proliferation, and reducing senescence‐associated gene and protein expression. ERK inhibition reduced senescence in control DFCs, while ERK activation enhanced senescence in mutant DFCs. The divergent effects of ERK on aging might depend on cellular context, where its activation can either delay or promote senescence based on specific regulatory networks and signaling dynamics.^[^ ^60^ ^]^ Moreover, inhibition of the Notch signaling pathway with DAPT significantly reduced the proliferation of human dental pulp cells and induced senescence, as evidenced by increased SA‐β‐gal staining. These findings suggest that Notch signaling plays a critical role in maintaining the proliferative capacity and delaying senescence of dental pulp cells.^[^ ^61^ ^]^ TGF‐β1 induces senescence in PDLSCs by elevating senescence‐associated β‐galactosidase activity and upregulating p16 and p21 expression. TGF‐β1 also promotes ROS production, while ROS scavengers reverse this effect, mitigating senescence.^[^ ^62^ ^]^ Proteomic analysis identified age‐correlated activation of the ILK/AKT/mTOR/STAT1 signaling pathway. The ILK inhibitor OSU‐T315 rejuvenates aged DPSCs by reducing senescence markers and enhancing osteoblastic differentiation and bone regeneration in vivo, offering a potential intervention for age‐related decline in DPSC function.^[^ ^63^ ^]^

### 5.2 Non‐Coding RNAs

Non‐coding RNAs, including microRNAs (miRNAs) and long non‐coding RNAs (lncRNAs), play pivotal roles in regulating the aging process of DSCs by modulating signaling pathways and gene network. Microarray analysis identified 27 senescence‐associated miRNAs in DPCs, with miR‐433 emerging as a regulator. MiR‐433 negatively modulates GRB2 and the RAS‐MAPK signaling pathway, impairing proliferation and mineralization while promoting apoptosis in DPCs. These findings suggest miR‐433 as a potential target for enhancing DPC repair and regeneration in aging.^[^ ^64^ ^]^ Similarly, aging in DPSCs is linked to differential expression of 14 mRNAs and 7 lncRNAs, with hsa‐miR‐6724‐5p identified as a regulator targeting genes involved in dopaminergic synapse pathways. These findings suggest a critical role for specific gene networks in DPSC senescence, highlighting potential molecular targets for mitigating age‐related decline in regenerative capacity.^[^ ^65^ ^]^ In addition, the upregulation of miR‐152 in DPSCs during senescence suppresses SIRT7 expression, promoting the development of a senescent phenotype. Targeted inhibition of miR‐152 or overexpression of SIRT7 effectively reverses senescence, suggesting this pathway as a promising target to improve DPSC functionality and therapeutic potential.^[^ ^66^ ^]^ Moreover, adrenomedullin (ADM) overexpression in DPSCs inhibits senescence by reducing ROS accumulation, suppressing p53, p21, and p16 expression, and promoting cell cycle progression. ADM achieves this by downregulating miR‐152, which targets CCNA2, thereby enhancing proliferation and resistance to aging.^[^ ^67^ ^]^ Notably, overexpression of miR‐141‐3p accelerates senescence and inhibits proliferation in SCAPs by post‐transcriptionally downregulating YAP. Conversely, miR‐141‐3p inhibition enhances proliferation and delays senescence, highlighting the miR‐141‐3p/YAP axis as a key regulator of SCAP aging and function.^[^ ^68^ ^]^

### 5.3 Epigenetic Regulation

**Figure 3:**
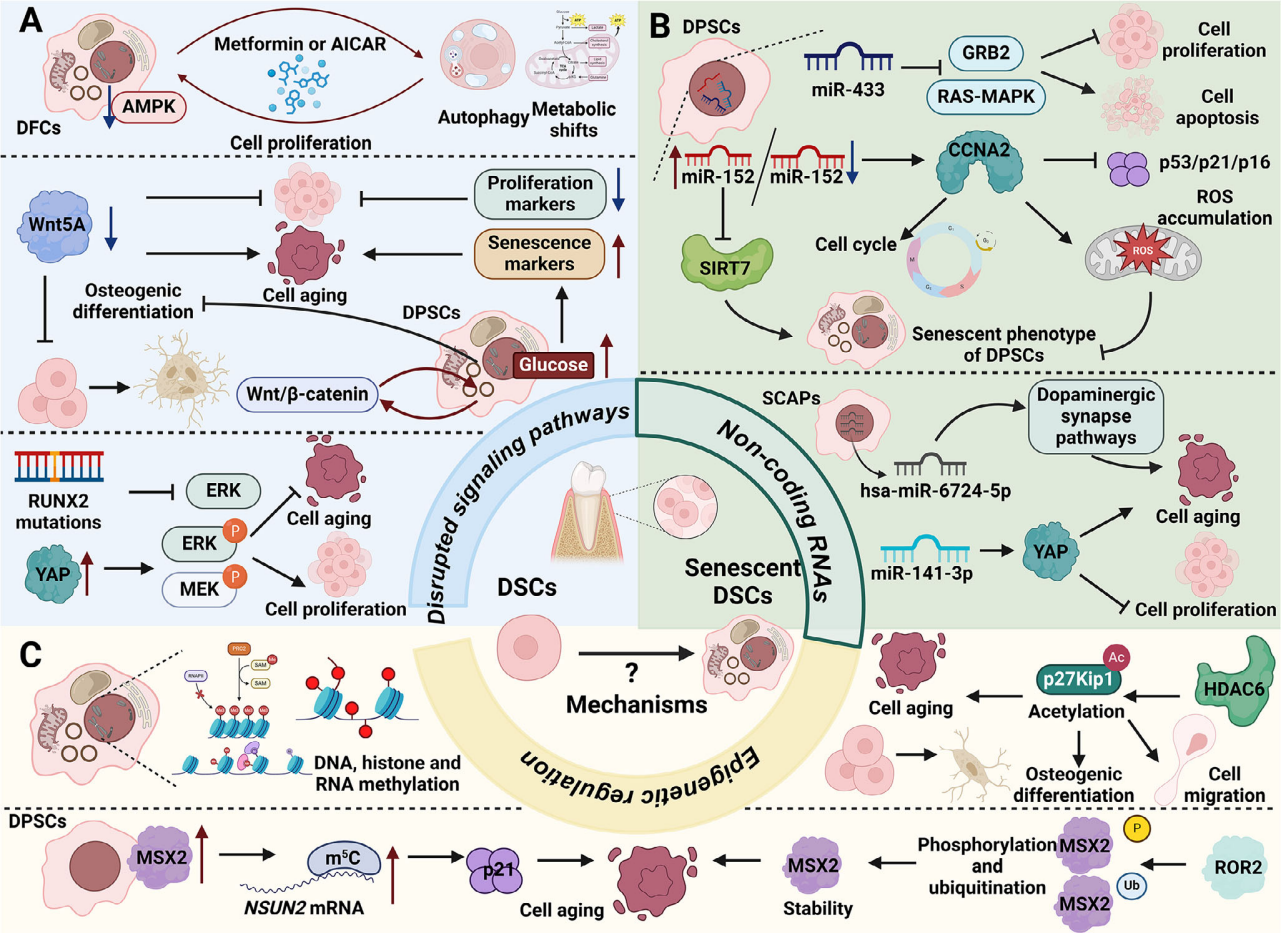
Molecular mechanisms of DSC aging. A) DSC senescence involves disrupted AMPK signaling, with metformin or AICAR modulating autophagy and glucose metabolism. Hyperglycemia accelerates aging via Wnt/β‐catenin, while Wnt5A supports osteogenic differentiation. RUNX2 mutations impair the ERK‐MEK pathway, promoting DPSC aging. B) miRNAs regulate GRB2‐RAS‐MAPK, affecting CCNA2 expression, ROS accumulation, cell cycle, and apoptosis. miR‐6724‐5p and miR‐141‐3p modulate senescence and proliferation via the dopaminergic pathway and YAP signaling. C) DNA, histone, and RNA methylation are key regulators of DSC aging. MSX2 phosphorylation and acetylation modulate osteogenic differentiation and aging, with ROR2 influencing MSX2 stability. HDAC6 regulates PDLSC aging by modulating p27Kip1 acetylation and stability.

Epigenetic regulation plays a pivotal role in DSC aging by orchestrating changes in DNA methylation, histone modifications, and RNA methylation. These alterations influence cellular processes, including differentiation, oxidative stress response, and senescence, highlighting their significance in maintaining stem cell functionality and therapeutic potential (**Figure** **3**). Replicative senescence in DPSC is associated with significant morphological changes and decreased Alu element methylation, with late‐passage cells showing elevated hypomethylated Alu CpG sites. These epigenetic alterations likely contribute to DPSC aging, impairing their regenerative potential and therapeutic efficacy.^[^ ^69^ ^]^ In addition, the ROR2/MSX2/NSUN2 axis plays a critical role in DPSC aging. Elevated MSX2 in aging DPSCs promotes senescence by upregulating NSUN2, which enhances p21 expression via 5‐methylcytidine methylation of its mRNA. MSX2 knockdown inhibits senescence, restoring self‐renewal, while ROR2 modulates MSX2 stability by affecting its phosphorylation and ubiquitination.^[^ ^70^ ^]^ Moreover, KDM3A enhances the chondrogenic differentiation capacity of aged DPSCs without affecting stemness. Elevated during senescence, KDM3A overexpression mitigates functional decline in aged DPSCs, highlighting its potential as a therapeutic target for rejuvenating stem cell function in cartilage regeneration.^[^ ^71^ ^]^ Advanced glycation end products (AGEs) impair osteogenic differentiation and antioxidative capacity of PDLSCs under mechanical force by disrupting KDM6B mechanical responsiveness. AGEs inhibit a KDM6B/Wnt feedback loop that regulates superoxide dismutase 2, leading to oxidative stress and stem cell aging. Targeting the AGE/RAGE pathway or enhancing KDM6B function may improve orthodontic outcomes in diabetic patients.^[^ ^72^ ^]^ Importantly, histone deacetylase 6 (HDAC6) regulates PDLSC aging by modulating p27Kip1 acetylation and stability. HDAC6 inhibition accelerates senescence, reducing osteogenic differentiation and migration, while elevating p27Kip1 levels. These findings highlight HDAC6 as a regulator of PDLSC function and aging.^[^ ^73^ ^]^

### 5.4 Metabolic Alteration

Metabolic alterations are integral to the aging process of DSCs, influencing their proliferative and regenerative capacities through multiple interconnected pathways. At the level of energy metabolism, PDLSCs undergoing replicative senescence display increased AMP:ATP ratios but paradoxically reduced AMPK phosphorylation and diminished FOXO1/FOXO3a expression, with partial restoration of AMPK activity upon pharmacological activation, underscoring impaired energy‐sensing as a hallmark of aging.^[^ ^74^ ^]^ One‐carbon metabolism and serine biosynthesis also play a pivotal role: aged DPSCs exhibit decreased expression of PSAT1 and PHGDH, leading to reduced S‐adenosylmethionine production, global hypomethylation, and upregulation of the senescence marker p16, while silencing PHGDH in young DPSCs similarly diminishes proliferation and osteogenic differentiation, highlighting the centrality of serine and one‐carbon units in maintaining stemness.^[^ ^75^ ^]^ Redox and NAD⁺ metabolism are likewise disrupted, as evidenced by elevated visfatin levels in aged dental pulp tissues and prematurely senescent pulp cells; visfatin knockdown attenuates senescence, whereas exogenous supplementation accelerates senescence through increased NADPH consumption, telomere damage, and NF‐κB‐driven SASP activation.^[^ ^76^ ^]^ Beyond these individual pathways, systems‐level analyses have revealed that metabolic flux profiling of DPSCs uncovers distinct signatures predictive of replicative senescence, enabling detection of functional decline before overt phenotypic changes, with Barx1 identified as a metabolic marker associated with this process.^[^ ^77^ ^]^ Together, these findings delineate a hierarchical framework of metabolic alterations—spanning energy sensing, one‐carbon metabolism, redox homeostasis, and global metabolic signatures—that collectively drive the aging trajectory of DSCs.

### 5.5 Other Critical Proteins Regulating DSC Aging

**Figure 4:**
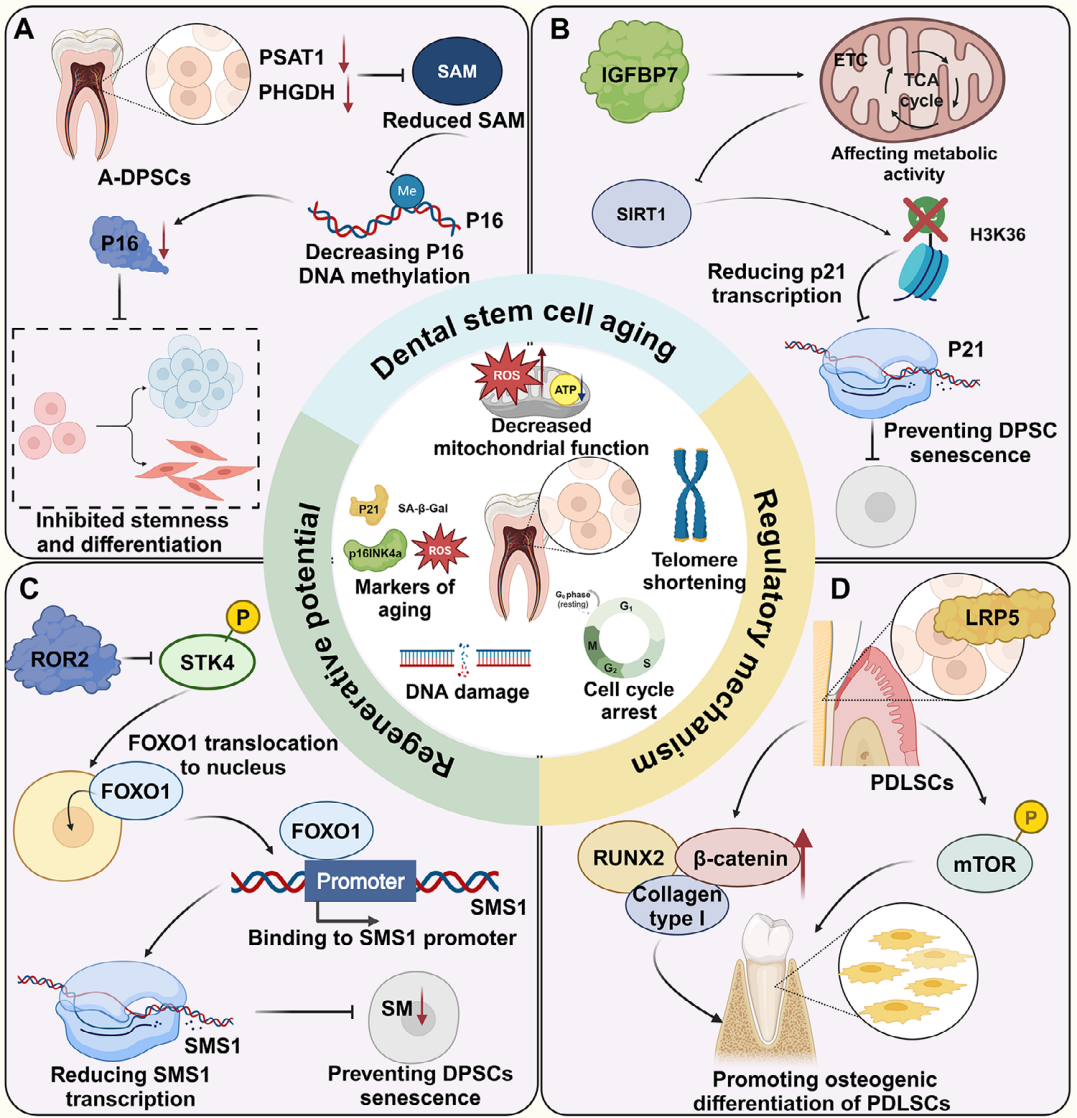
Additional regulators of DSC aging. A) In aging‐DPSCs, reduced PSAT1/PHGDH expression lowers SAM levels, decreasing p16 DNA methylation and upregulating p16, leading to diminished stemness and differentiation. B) IGFBP7 activates SIRT1 via metabolic pathways, inducing H3K36 deacetylation and suppressing p21 transcription, thereby delaying DPSC aging. C) ROR2 inhibits STK4 phosphorylation, promoting FOXO1 nuclear translocation. FOXO1 binds the SMS1 promoter, reducing SMS1 transcription, which decreases SM biosynthesis and prevents DPSC senescence. D) LRP5 upregulates RUNX2, β‐catenin, and COL1A1 in PDLSCs, while phosphorylated mTOR enhances nuclear accumulation, promoting osteogenic differentiation.

The regulation of DSC aging involves a network of critical proteins that influence cellular processes (**Figure** **4**). Single‐cell RNA sequencing of young and aged dental pulp revealed reduced fibroblast proportions and enhanced intercellular communication in aged tissue, particularly through FGF and IGF signaling pathways. IGFBP7, significantly upregulated in aged dental pulp, was shown to mitigate senescence in H_2_O_2_‐induced dental pulp fibroblasts. Additionally, IGFBP7 prevents senescence and enhances osteogenic differentiation of DPSCs by activating SIRT1‐mediated deacetylation of H3K36ac, reducing p21 transcription. Mitochondrial ETC activation by coenzyme Q10 rescues senescence induced by IGFBP7 knockdown, highlighting its role in maintaining DPSC function and its potential as a target for tissue regeneration in aging environments.^[^ ^78^ ^]^ Beyond IGFBP7, the SIRT family also plays a crucial role in regulating DSC aging. SIRT deficiency accelerates senescence and impairs osteogenic differentiation of PDLSCs under diabetes‐associated periodontitis. SIRT3 deacetylates LRPPRC to regulate oxidative phosphorylation and oxidative stress, mitigating senescence. Activation of SIRT3 by honokiol delayed senescence and promoted alveolar bone regeneration in diabetic mice, highlighting SIRT3 as a potential therapeutic target for diabetes‐related periodontal disease.^[^ ^79^ ^]^ Additionally, the SIRT7/ROCK1 axis regulates DPSC senescence by desuccinylating ROCK1 at K520. Reduced SIRT7 expression in replicative senescence increases senescence markers and decreases TERT activity, while ROCK1 inhibition reverses SIRT7 knockdown effects. Targeting this pathway may enhance DPSC therapeutic potential in aging contexts.^[^ ^80^ ^]^

Proteomic analysis of DPSCs from elderly donors revealed senescence‐associated changes, including upregulated HMGN1, HMGN2, UCHL1, and FAM96B. FAM96B depletion increased senescence markers and reduced TERT activity, while its overexpression enhanced proliferation, osteogenic differentiation, and mineralization potential. In addition, the ROR2/STK4‐FOXO1/SMS1 axis regulates sphingomyelin (SM) biogenesis and DPSC senescence. Reduced ROR2 expression in aged DPSCs impairs self‐renewal by promoting FOXO1 nuclear translocation and SMS1 repression, which inhibits SM production. ROR2 overexpression or STK4 inhibition restores DPSC proliferation, highlighting a potential target to combat tooth aging.^[^ ^81^ ^]^ Notably, aging and cellular senescence exacerbate periodontitis through the SASP, impairing PDLSC function. Procyanidin B2 mitigates these effects by reducing inflammation, enhancing antioxidant enzyme activity, and promoting osteogenic differentiation of PDLSCs under *Porphyromonas gingivalis* lipopolysaccharide‐induced inflammation.^[^ ^82^ ^]^ Low‐density lipoprotein receptor‐related protein 5 (LRP5) enhances osteogenic differentiation in normal and aged PDLSCs by upregulating RUNX2, collagen type I, and β‐catenin levels, and activating mTOR signaling. LRP5 overexpression partially restores osteogenic capacity in aged PDLSCs, while mTOR inhibition reverses this effect.^[^ ^83^ ^]^ Interestingly aging‐associated progerin accumulation activates endoplasmic reticulum (ER) stress in PDLSCs, suppressing stemness via the ATF6/p53/p21 axis. Inhibition of ER stress restores stemness in aged PDLSCs, highlighting the role of ER stress and the unfolded protein response in age‐related decline of regenerative potential. Short telomeres and DNA damage drive senescence in DFCs, with the DDR playing a role. Downregulation of DDR genes coincided with double‐strand breaks and senescence induction. DNA‐PK inhibition reduced ROS and senescence but impaired osteogenic differentiation, glycolysis, and proliferation, indicating cellular exhaustion. These findings highlight DNA‐PK as a promoter of senescence, while its inhibition fails to rejuvenate senescent DFCs.

## 6 Strategies to Mitigate DSC Aging for Enhanced Regenerative Potential

**Figure 5:**
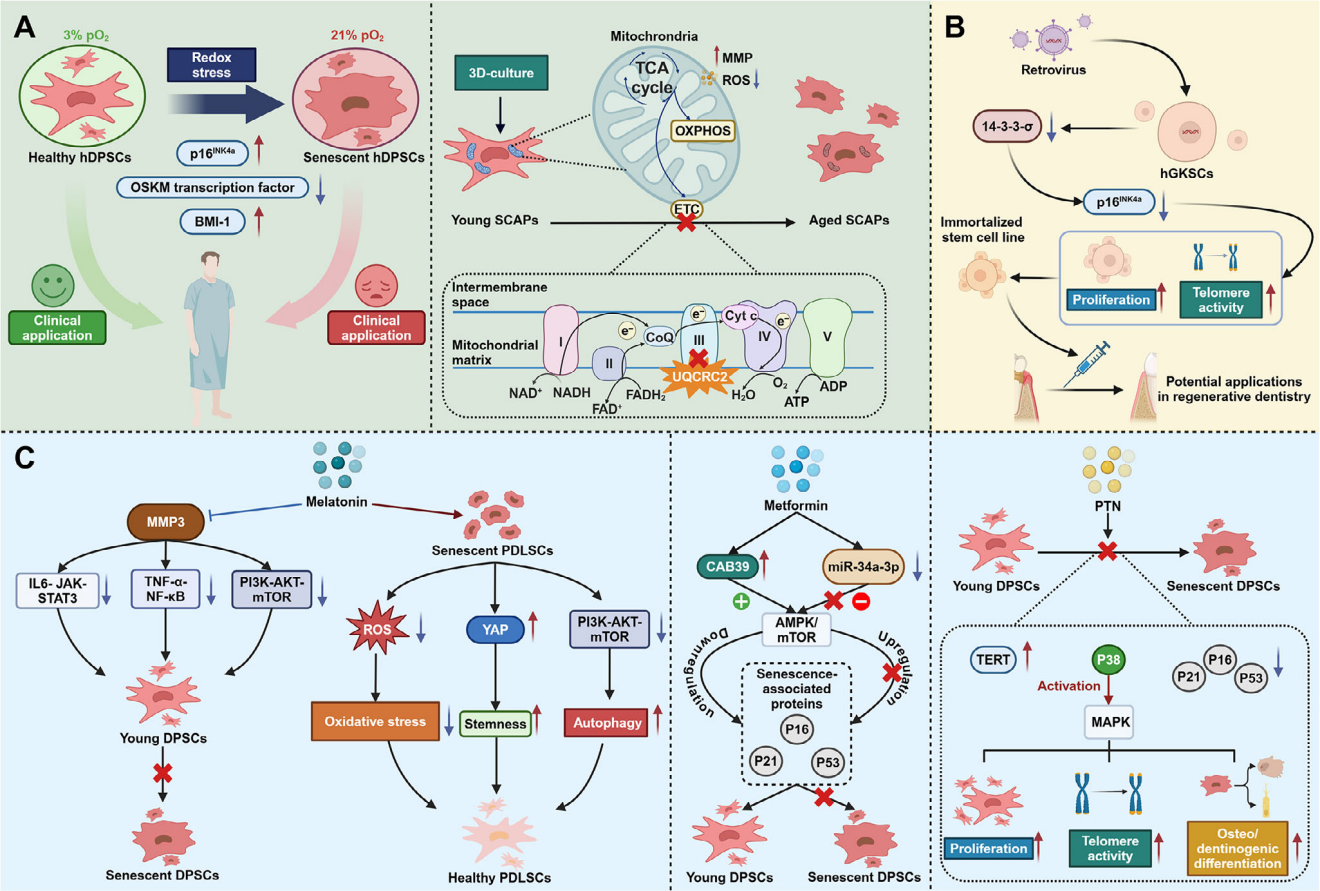
Strategies to mitigate DSC aging. A) Under ambient O_2_, DPSCs undergo oxidative stress and senescence via p16, whereas physiological O_2_ preserves stemness by upregulating OSKM and BMI‐1. In 3D culture, SCAPs maintain mitochondrial homeostasis and delay senescence via UQCRC2 regulation. B) Stable 14‐3‐3σ downregulation immortalizes hGKSCs, enhancing proliferation and telomere activity, supporting regenerative dental applications. C) Melatonin inhibits MMP3, preventing DPSC senescence via IL6‐JAK‐STAT3, TNF‐α‐NF‐κB, and PI3K‐AKT‐mTOR pathways. It also reduces ROS in PDLSCs, promoting YAP expression. Metformin suppresses miR‐34a‐3p, upregulates CAB39, and activates AMPK/mTOR, reducing senescence markers. PTN protects DPSCs from senescence via p38 MAPK, enhancing telomere activity and osteogenic/dentinogenic differentiation.

Strategies to enhance the functionality and mitigate the senescence of dental DSCs are essential for maximizing their therapeutic potential in regenerative medicine. Approaches such as optimizing culture conditions, supplementing with exogenous substances, and employing genetic engineering techniques offer significant potential to overcome age‐related limitations, paving the way for more effective stem cell‐based therapies (**Figure** **5**, **Table** **2**). Long‐term culture of DPSCs at ambient oxygen tension (21%) induces oxidative stress‐related senescence via the p16 pathway. In contrast, culturing DPSCs at physiological oxygen tension (3–6%) preserves stemness and delays senescence, highlighting the critical role of optimizing culture conditions for maintaining their therapeutic potential in clinical applications.^[^ ^84^ ^]^ In addition, 3D culture conditions delay senescence in SCAPs by preserving mitochondrial homeostasis through UQCRC2‐mediated regulation of oxidative phosphorylation.^[^ ^85^ ^]^ Importantly, hypoxia‐preconditioned 3D culture‐derived extracellular vesicles from GMSCs enhance proliferation, reduce senescence, and restore mitochondrial dynamics in aging GMSCs. Transcriptomic analysis revealed upregulation of cell cycle, DNA repair, and mitochondrial function genes, offering a novel strategy to mitigate senescence and optimize GMSC production for clinical use.^[^ ^86^ ^]^ The addition of exogenous substances from various sources represents an effective strategy for reversing DSC aging. Melatonin mitigates DPSC senescence induced by long‐term expansion by inhibiting MMP3 expression. MMP3 overexpression reverses this anti‐senescent effect, linking its role to pathways including IL6‐JAK‐STAT3, TNF‐α‐NF‐κB, and PI3K‐AKT‐mTOR. These findings highlight MMP3 as a key regulator of DPSC aging and the protective effects of melatonin.^[^ ^87^ ^]^ Similarly, melatonin mitigates oxidative stress‐induced senescence in PDLSCs by reducing ROS accumulation, preserving stemness, and enhancing YAP expression. MT delays aging and maintains regenerative potential in an inflammatory microenvironment.^[^ ^88^ ^]^ Long‐term expansion of PDLSCs induces senescence and impairs autophagy. Melatonin reverses these effects by restoring autophagic processes via the melatonin‐dependent PI3K/AKT/mTOR signaling pathway. Melatonin supplementation rejuvenates senescent cells, highlighting its potential as an autophagy‐restoring agent for optimizing clinical‐scale stem cell production protocols.^[^ ^89^ ^]^ Notably, metformin mitigates DPSC senescence by suppressing miR‐34a‐3p, upregulating CAB39, and activating the AMPK/mTOR pathway. This reduces senescence markers, enhances proliferation, and promotes resistance to aging, positioning metformin as a promising agent for improving DPSC‐based regenerative therapies.^[^ ^90^ ^]^ Curcumin mitigates replicative senescence in DFCs by downregulating senescence markers and restoring proliferation and osteogenic differentiation markers. At 50 µM, curcumin inhibits senescence and enhances proliferation without altering cell size, suggesting its potential as an anti‐senescence therapeutic to preserve DFC function and osteogenic capacity.^[^ ^45^ ^]^ Notably, quercetin modulates mitochondrial activity in SHEDs in a dose‐ and age‐dependent manner. It enhances metabolic activity and mitochondrial respiration in younger SHEDs but supports mitochondrial function at lower doses in senescent SHEDs, while higher doses inhibit respiration. These findings highlight the potential of quercetin in maintaining SHED viability and function, particularly in aging cells, with implications for optimizing its use in regenerative medicine.^[^ ^91^ ^]^ Notably, hydrogen sulfide (H_2_S) alleviates senescence in PDLSCs by activating TRPV4‐mediated calcium influx, promoting cell proliferation, and reducing the expression of senescence markers. These findings highlight H_2_S as a potential therapeutic strategy to combat cellular aging and enhance stem cell‐based treatments for oral diseases.^[^ ^92^ ^]^ CCL11 antibody enhances pulp regeneration in aged mice by reducing blood CCL11 levels and shifting macrophage polarization toward an M2 phenotype, decreasing the M1/M2 ratio. These findings suggest that targeting CCL11 improves the stem cell niche and promotes dental pulp regeneration in aging.^[^ ^93^ ^]^ Interestingly, CCR3 antagonist (CCR3A) mitigates p‐Cresol‐induced senescence in PDLSCs by reducing senescence markers, suppressing CCL11, and enhancing IDO expression. In aged dog teeth, CCR3A improves pulp regeneration by promoting neurite extension, migratory activity, and anti‐inflammatory effects, highlighting its potential to rejuvenate dental tissues and combat age‐related decline.^[^ ^94^ ^]^ Brain‐derived neurotrophic factor (BDNF) alleviates senescence in PDLSCs by inhibiting Notch3, enhancing osteogenic differentiation, and reducing senescence markers. During orthodontic tooth movement, exogenous BDNF promotes osteogenesis and mitigates age‐related delays, highlighting its therapeutic potential in aging‐related orthodontic challenges.^[^ ^95^ ^]^ Similarly, pleiotrophin (PTN) protects DPSCs from senescence by enhancing proliferation, telomere activity, and osteo/dentinogenic differentiation via the p38 MAPK pathway. PTN depletion increases senescence markers and reduces TERT expression, while recombinant PTN reverses these effects, mitigating age‐related functional decline in DPSCs.^[^ ^96^ ^]^ Treatment with AEDG and KED peptides significantly reduced p16 and p21 mRNA levels in PLDSCs and GMSCs (1.56–3.23‐fold) and preserved cell morphology, as confirmed by immunofluorescence. These peptides exhibit geroprotective properties and hold promise for enhancing large‐scale stem cell expansion for clinical applications.^[^ ^97^ ^]^ ZF1, a zebrafish embryo extract, modulates senescence in MSCs from various tissues, including DP‐MSCs, by reducing β‐galactosidase activity, enhancing TERT expression, and inducing BMI1. It downregulates senescence repressors TP53 and CDKN1A, suggesting its potential to modulate stem cell senescence and enhance regenerative capacity in vitro and in vivo.^[^ ^98^ ^]^ Genetic engineering of key genes associated with DSC aging is also a promising strategy. Stable down‐regulation of 14‐3‐3 sigma protein enabled the creation of an immortalized human gingival keratinocyte stem cell line, significantly enhancing proliferation capacity and maintaining over 90% stem cell content. This approach extended culture lifespan to 110 doublings compared to controls. These cells offer a stable platform for stem cell differentiation studies, gene therapy targeting gingival epithelium, and testing oral hygiene products, with potential applications in regenerative dentistry and preprosthetic surgery.^[^ ^99^ ^]^

**Table 2:** Strategies to mitigate DSC aging.

Strategy	Effect on Senescence	Targeted Agents	Targeted Cell Type	Potential Application	Refs.
Optimizing culture conditions	Induces oxidative stress‐related senescence	p16 pathway	DPSCs SCAPs	Enhancing regenerative potential in clinical applications	[84]
3D Culture conditions	Delays senescence by preserving mitochondrial homeostasis	UQCRC2‐mediated oxidative phosphorylation	SCAPs GMSCs	Optimizing DSC culture for dental tissue regeneration	[85]
Hypoxia‐preconditioned 3D Culture	Enhance proliferation, reduce senescence, and restore mitochondrial dynamics	Mitochondrial function genes	GMSCs	Mitigating senescence in aging DSCs, optimizing DSC production for clinical use	[86]
Melatonin supplementation	Mitigates senescence induced by long‐term expansion	MMP3	DPSCs PDLSCs	Anti‐senescence, autophagy restoration of DSC	[87]
Melatonin supplementation	Reduces senescence markers and enhances proliferation	AMPK/mTOR	DPSCs	Improving regenerative therapies for dental tissues	[90]
Curcumin supplementation	Mitigates replicative senescence, restores proliferation	Senescence markers	DFCs	Preserving DSC function and osteogenic capacity	[45]
Quercetin supplementation	Modulates mitochondrial activity	Mitochondrial respiration	SHEDs	Maintaining DSC viability and function, particularly in aging cells	[91]
H_2_S treatment	Alleviates senescence	TRPV4‐mediated calcium influx	PDLSCs	Combating cellular aging, enhancing DSC‐based treatments for oral diseases	[92]
CCL11 antibody	Enhances pulp regeneration	Macrophage polarization	DPSCs GMSCs	Enhancing DSC niche, improving dental pulp regeneration in aging	[93]
CCR3A supplementation	Mitigates senescence	CCL11 IDO	PDLSCs	Rejuvenating dental tissues, combating age‐related decline	[94]
BDNF supplementation	Alleviates senescence	Notch3	PDLSCs	Enhancing orthodontic tooth movement, combating age‐related delays	[95]
PTN supplementation	Protects DPSCs from senescence, enhances proliferation	p38 MAPK pathway	DPSCs	Mitigating age‐related decline, promoting regenerative capabilities	[96]
AEDG and KED peptides supplementation	Preserves cell morphology, exhibits geroprotective properties	p16 and p21 mRNA	PLDSCs GMSCs	Enhancing large‐scale DSC expansion for clinical applications	[97]
ZF1 supplementation	Modulates senescence in DSCs from various tissues	↓ β‐galactosidase ↑ TERT ↑ BMI1	DP‐MSCs	Enhancing regenerative capacity in DSCs, modulating senescence in vitro and in vivo	[98]
14‐3‐3 sigma downregulation	Enhances proliferation, extends culture lifespan	/	hGKSCs	Creating immortalized DSC lines for regenerative dentistry, gene therapy	[99]

Importantly, the molecular pathways implicated in DSC aging have direct relevance to age‐related oral diseases. In periodontitis, senescent PDLSCs with impaired Wnt and TGF‐β signaling exhibit diminished osteogenic differentiation and enhanced SASP activity, fostering a chronic inflammatory milieu that accelerates alveolar bone resorption and periodontal tissue destruction.^[^ ^100^ ^]^ In the pulp–dentin complex, age‐associated activation of Wnt/β‐catenin and Notch pathways in DPSCs reduces proliferative and odontogenic potential, predisposing to pulp fibrosis and impaired reparative dentinogenesis in elderly individuals.^[^ ^101^ ^]^ Moreover, metabolic alterations such as impaired serine biosynthesis and elevated visfatin exacerbate oxidative stress and telomere instability, processes that overlap with the pathogenesis of diabetes‐associated periodontitis and delayed wound healing. Together, these connections highlight that the same signaling disruptions driving stem cell senescence also accelerate the onset and progression of age‐related oral disorders, underscoring the importance of integrating molecular insights with clinical strategies for prevention and regeneration.

## 7 Challenges and Perspectives

**Figure 6:**
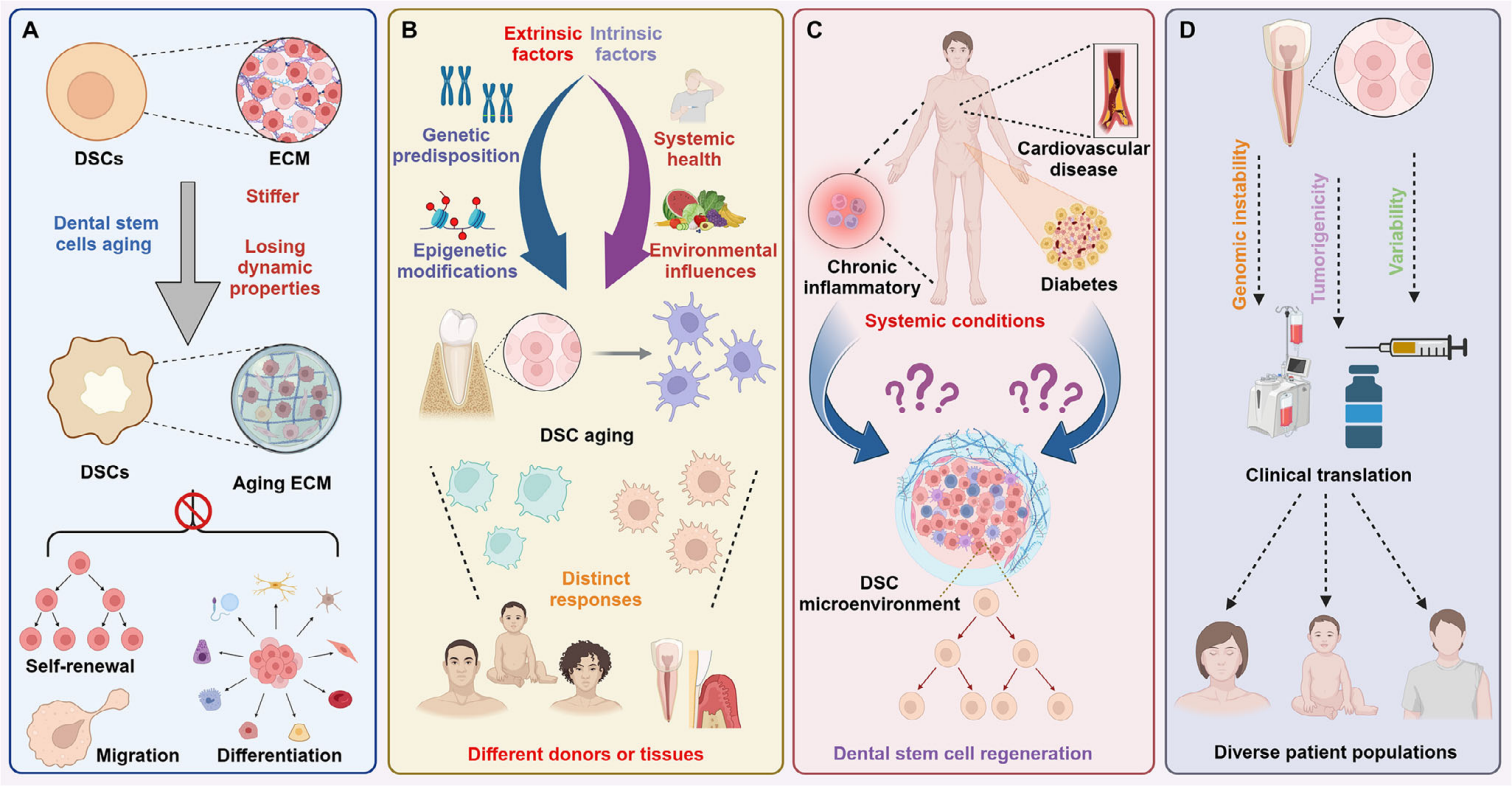
Challenges and perspectives of DSC aging in regenerative therapies. A) Aging increases ECM stiffness and reduces its dynamic properties, impairing DSC self‐renewal, migration, and differentiation, ultimately decreasing regenerative potential. B) DSC aging is influenced by intrinsic factors (genetics, epigenetics) and extrinsic factors (systemic health, environment). Additionally, DSC aging varies across individuals and tissues. C) Systemic diseases (oxidative stress, inflammation, metabolic dysregulation) accelerate DSC aging by altering the DSC microenvironment. However, the molecular mechanisms remain unclear. D) Challenges such as genomic instability, tumorigenicity, and therapeutic variability limit DSC clinical translation, necessitating further research on long‐term safety and patient‐specific adaptations.

Aging in DSCs is governed by a multifaceted interplay of intrinsic and extrinsic factors, presenting unique challenges in understanding and mitigating functional decline. Below, we highlight key challenges and propose integrated strategies that hold promise for advancing regenerative dentistry and stem cell‐based therapeutics (**Figure** **6**).

First, aging significantly alters the DSC niche by modifying the composition and mechanical properties of the ECM and disrupting intercellular signaling. The aging ECM often becomes stiffer and loses its dynamic properties, while the microenvironment shifts toward a pro‐inflammatory and oxidative state. These changes impair DSC self‐renewal, migration, and differentiation, contributing to the decline in regenerative capacity.^[^ ^102^ ^]^ Despite the recognized importance of the ECM in stem cell function, the precise mechanisms by which these alterations drive aging remain poorly understood. Leveraging advancements in bioengineering, functionalized biomaterials such as hydrogels or bioactive scaffolds can be designed to mimic the native ECM while counteracting the aging microenvironment. These materials can be embedded with anti‐inflammatory agents, antioxidant molecules, or growth factors to promote cell survival and proliferation. Additionally, spatiotemporal control of bioactive molecule release through engineered nanoparticle systems can provide targeted support to DSCs.^[^ ^103^ ^]^

Second, DSCs display considerable heterogeneity in their aging trajectories and regenerative capacities, influenced by intrinsic factors such as genetic predisposition and epigenetic modifications, as well as extrinsic factors including systemic health conditions (e.g., diabetes, chronic inflammation) and environmental influences such as diet and lifestyle. Specifically, single‐cell RNA‐seq with flow‐cytometric validation now documents a perivascular MCAM(+)JAG(+)PDGFRA(–) hDPSC subset that persists at ≈2% of total pulp cells across physiological aging and caries. In addition, clonal analyses (DF2/DF8/DF18) quantify chromosomal instability at the sub‐clone level and show that three‐clone co‐culture reduces the aneuploidy ratio versus single‐clone cultures while partially preserving osteogenic capacity. These endpoints (subset frequency, aneuploidy ratio, differentiation indices) provide measurable evidence for ′considerable heterogeneity.^[^ ^104^ ^]^ This variability complicates the application of standardized therapeutic strategies, as interventions effective in one subset of patients may fail in others. Recent updates from the International Society for Cell and Gene Therapy (ISCT) have emphasized a more precise distinction between mesenchymal stromal cells and mesenchymal stem cells, underscoring their heterogeneous nature and clinical reporting requirements.^[^ ^105^ ^]^ Within the dental field, most dental‐derived cell populations—including pulp, periodontal ligament, apical papilla, and exfoliated deciduous teeth—exhibit mesenchymal stromal/stem cell–like features, while the dental follicle has now been recognized as another important mesenchymal stromal cell source.^[^ ^106^ ^]^ In this context, it may be more appropriate to consider dental‐derived cells as tissue‐specific stromal cell populations rather than uniformly categorizing them as stem cells. This perspective not only aligns dental‐derived stem/progenitor cells with the broader mesenchymal stromal cell framework but also highlights potential differences in their developmental plasticity, niche‐specific properties, and immunomodulatory functions, which may bear significance for both fundamental studies and translational applications.

Moreover, DSCs from different donors or tissues may exhibit distinct responses to the same aging stimuli or anti‐aging interventions, further underscoring the need for personalized approaches. To address this complexity, single‐cell multi‐omics technologies, including transcriptomics, proteomics, and epigenomics, can be employed to map the molecular heterogeneity of DSCs in both young and aged populations.^[^ ^107^ ^]^ These high‐resolution datasets enable the identification of specific molecular signatures or pathways driving aging in individual cells, facilitating the development of personalized treatment regimens. For example, targeted therapies could focus on correcting metabolic imbalances or epigenetic dysregulation unique to a particular subset of aging DSCs. Biomarker discovery plays a pivotal role in this process, with efforts aimed at identifying secreted proteins, non‐coding RNAs, or metabolic products that correlate with specific aging states. These biomarkers could serve as diagnostic tools for early detection of functional decline or as therapeutic targets to reverse aging‐related impairments. Furthermore, patient‐derived organoids—3D cell cultures that replicate key features of native tissues—offer a robust preclinical model to evaluate individual responses to anti‐aging interventions. By integrating organoid technology with patient‐specific data, researchers can simulate treatment outcomes and refine therapeutic strategies before clinical application, ensuring greater efficacy and safety.^[^ ^4^, ^108^ ^]^

Third, systemic conditions, such as diabetes, cardiovascular disease, and chronic inflammatory disorders, significantly accelerate the aging of DSCs. These conditions exacerbate oxidative stress, chronic inflammation, and metabolic dysregulation, which disrupt the delicate balance of the DSC microenvironment and impair regenerative capacity. Despite these recognized connections, the precise molecular crosstalk between systemic diseases and local DSC aging remains underexplored, hindering the development of targeted and effective therapeutic strategies.^[^ ^109^ ^]^ Employing single‐cell transcriptomics and proteomics in both local and systemic tissues can uncover the molecular pathways linking systemic diseases to DSC dysfunction.^[^ ^110^ ^]^ This includes identifying circulating biomarkers, such as cytokines, metabolic byproducts, or oxidative stress markers, which may mediate cross‐organ communication and drive DSC aging. For instance, in type 2 diabetes, DPSCs show diminished clonogenicity, reduced osteogenic and chondrogenic potential, and impaired angiogenesis, while exhibiting enhanced adipogenic bias, reflecting hyperglycemia‐driven lineage imbalance. Under chronic inflammatory conditions, sustained TNF‐α exposure activates NF‐κB and VEGF signaling, leading to telomere shortening, chromosomal instability, and impaired mineralization capacity—effects that can be partially rescued by NF‐κB inhibition.^[^ ^111^ ^]^ Complementary proteomic profiling of DFSCs and SCAP further highlights differential expression of proteins involved in autophagy regulation and immune stability (e.g., MARCKS, NPC1), suggesting that systemic diseases reshape DSC functions through convergent pathways such as NF‐κB, VEGF, telomere maintenance, and autophagy.^[^ ^112^ ^]^ Additionally, complementary to addressing systemic effects, strategies to bolster DSC resilience locally are essential. ROS scavengers or mitochondrial‐targeted antioxidants, such as mitoTEMPO, could reduce oxidative stress within DSCs.^[^ ^113^ ^]^ Additionally, localized delivery of anti‐inflammatory agents, such as corticosteroids or NF‐κB inhibitors, via hydrogels or nanoparticles may protect DSCs from systemic inflammatory burden.^[^ ^114^ ^]^

Despite the potential of DSC‐based therapies, clinical translation faces significant hurdles, including the risks of genomic instability, tumorigenicity, and variability in therapeutic outcomes. The long‐term safety of gene editing, biomaterials, and pharmacological interventions in aging contexts requires rigorous evaluation.^[^ ^115^ ^]^ Furthermore, the scalability of such therapies remains a challenge, especially when applied to diverse patient populations with variable aging rates and health conditions. Multimodal approaches combining biomaterials, gene editing, and drug delivery systems can address these challenges by enhancing both safety and efficacy. For instance, biocompatible scaffolds integrated with controlled‐release systems for growth factors or small molecules can localize therapeutic effects and minimize off‐target risks. Predictive modeling using artificial intelligence can optimize treatment parameters, such as dosage and timing, to improve consistency across patient populations. Additionally, long‐term preclinical studies are essential to assess the durability and safety of these interventions in aging contexts.^[^ ^116^, ^117^, ^118^ ^]^

Beyond the biological and translational barriers, technical challenges critically constrain the advancement of DSC‐based therapies. A major issue lies in the efficiency and reproducibility of stem cell isolation and purification. Current methods often yield heterogeneous cell populations due to variability in tissue sources, donor age, and systemic health conditions, which complicates both basic research reproducibility and the establishment of standardized therapeutic protocols.^[^ ^119^ ^]^ Equally important is the stability of stem cells during large‐scale culture and expansion, as prolonged in vitro propagation frequently results in phenotypic drift, accelerated senescence, and diminished multipotency. These changes not only impair regenerative potential but also increase the risk of inconsistent therapeutic outcomes.^[^ ^120^ ^]^ Moreover, scaling up production under GMP conditions requires stringent control of cell quality, including genomic stability, immunophenotype, and functional potency, which remains technically demanding. Addressing these challenges will require the development of refined isolation protocols, advanced biomaterial scaffolds, and bioreactor‐based culture systems that better mimic the native microenvironment, as well as the incorporation of real‐time quality control technologies. Such innovations are essential to ensure both the reliability of preclinical research and the scalability and safety of clinical applications.

## 8 Conclusion

Aging imposes profound challenges on the regenerative potential of DSCs, impairing their proliferation, differentiation, and immunomodulatory functions. Accumulating evidence demonstrates that intrinsic mechanisms such as genomic instability, mitochondrial dysfunction, epigenetic dysregulation, and metabolic imbalance, together with extrinsic influences including systemic inflammation and niche deterioration, converge to drive DSC senescence. These alterations not only limit their utility in tissue regeneration but also compromise their ability to maintain homeostasis within the oral and systemic microenvironment. Recognizing the multifactorial nature of DSC aging underscores the importance of integrated strategies that target both cellular mechanisms and microenvironmental support systems.

Recent advances provide encouraging prospects for mitigating age‐related decline in DSCs. Approaches such as modulation of Wnt/β‐catenin or AMPK/mTOR signaling, restoration of telomere function, metabolic reprogramming, and epigenetic remodeling have demonstrated efficacy in preclinical settings.^[^ ^121^, ^122^, ^123^ ^]^ Moreover, exogenous interventions‐including melatonin, metformin, and antioxidants—as well as extracellular vesicles derived from young or preconditioned stem cells, show promise in rejuvenating aged DSCs and restoring their regenerative functions.^[^ ^87^, ^90^ ^]^ Bioengineered scaffolds and niche‐modulating biomaterials further expand the therapeutic toolkit, enabling the design of microenvironments that counteract aging‐associated dysfunction.^[^ ^124^ ^]^ Together, these strategies reveal a dynamic landscape where molecular biology, biomaterials science, and pharmacology converge to extend the functional lifespan of DSCs.

Despite this progress, several unresolved issues warrant careful consideration. Donor heterogeneity and inter‐individual variability remain major obstacles, as DSCs from different sources or systemic contexts display divergent aging trajectories and therapeutic responses. Furthermore, systemic conditions such as diabetes, cardiovascular disease, and chronic inflammation accelerate DSC aging and reduce therapeutic efficacy, highlighting the need for holistic, patient‐specific treatment strategies. Safety concerns—including genomic instability, tumorigenicity, and long‐term durability‐pose additional barriers to clinical translation. Addressing these challenges will require the integration of high‐resolution multi‐omics profiling, patient‐derived organoid models, and predictive computational approaches to enable personalized and adaptive interventions.

Looking ahead, a multidisciplinary framework is essential to unlock the full therapeutic potential of DSCs in aging populations. Advances in stem cell biology can uncover molecular targets for rejuvenation, while bioengineering and biomaterials provide supportive niches that restore functionality. Nanomedicine enables precise delivery of antioxidants, gene modulators, or signaling regulators, and artificial intelligence can integrate multi‐omics data to optimize individualized treatment strategies. To ensure safe translation, rigorous preclinical validation and carefully designed clinical trials are required, with particular attention to genomic stability, immunogenicity, and long‐term efficacy. Ultimately, DSC‐based regenerative medicine holds promise not only for restoring oral health in elderly individuals but also for establishing a broader paradigm to combat age‐related degeneration across multiple organ systems.

## Conflict of Interest

The authors declare no conflict of interest.

## Author Contributions

X.Z. and Y.F.L contributed equally to this work. L.C. and X.Z. conceptualized the idea of the review. L.C., Y.F.L., and P.L. prepared initial drafts of the manuscript. L.C., X.Z., Y.F.L., L.P., and Y.L., X.C., and Z.Z.H. contributed to the writing, graph creation and manuscript improvement. L.C., X.Z., Y.F.L., L.P., and Y.L., X.C., and Z.Z.H. contributed to the creation of graphs, tables. All authors reviewed the manuscript and approved to the final version of this manuscript.
